# PPARγ gene C161T substitution alters lipid profile in Chinese patients with coronary artery disease and type 2 diabetes mellitus

**DOI:** 10.1186/1475-2840-9-13

**Published:** 2010-03-24

**Authors:** Jing Wan, Shixi Xiong, Shengping Chao, Jianming Xiao, Yexin Ma, Jinghua Wang, Sabita Roy

**Affiliations:** 1 Department of Cardiology, Zhongnan Hospital of Wuhan University, Wuhan, 430071, China; 2 Department of Cardiology, Tongji Hospital of Huazhong Science and Technology University, Wuhan, 430030, China; 3 Division of Basic and Translational Research, Department of Surgery, University of Minnesota, Minneapolis, MN 55455, USA

## Abstract

**Background:**

Peroxisome proliferator-activated receptor γ (PPARγ) is a ligand-activated transcription factor, which regulates gene expression of the key proteins involved in lipid metabolism, vascular inflammation, and proliferation. PPARγ may contribute to attenuating atherogenesis and postangioplasty restenosis. PPARγ C161→T substitution is associated with a reduced risk of coronary artery disease (CAD). Whether or not the gene substitution alters the risk of CAD in type 2 diabetes mellitus (T2DM) patients remains unclear.

**Methods:**

A total of 556 unrelated subjects from a Chinese Han population, including 89 healthy subjects, 78 CAD patients, 86 T2DM patients, and 303 CAD combined with T2DM patients, were recruited to enroll in this study. PPARγC161→T gene polymorphism was determined by polymerase chain reaction and restriction fragment length polymorphisms. Plasma levels of lipoproteins, apolipoproteins, glucose, and insulin were measured by ELISA or radioimmunoassay (RIA). The coronary artery lesions were evaluated by coronary angiography.

**Results:**

The frequency of the 161T allele in CAD, T2DM, and CAD combined with T2DM patients was similar to that observed in the healthy control group. However, in CAD combined with T2DM patients, the group with angiographically documented moderate stenoses had a higher frequency of the 161T allele in comparison to the group with severe stenoses (P < 0.05). Moreover, in CAD with T2DM patients, the triglyceride levels and apoB in CC homozygote carriers were significantly higher than those in "T" allele carriers.

**Conclusions:**

PPARγC161→T genotypes weren't significantly associated with the risk of CAD, but were markedly correlated with severity of disease vessels in patients with CAD and T2DM. Furthermore, PPARγC161→T substitution was associated with an altered adipose, but not glucose metabolism. These results indicate that the PPARγ C161→T polymorphism may reduce the risk of severe atherogenesis by modulation of adipose metabolism, especially triglycerides and apoB, in Chinese patients with CAD and T2DM.

## Background

Peroxisome proliferation-activated receptor (PPAR) is a family of ligand-activated transcription factors, which has three isotypes, namely α, β and γ [1,2]. It has been demonstrated that PPARγ plays important roles in controlling lipid and glucose metabolism, and is currently known to be implicated in various metabolic diseases such as hyperlipidemia, diabetes mellitus, and coronary artery disease (CAD) [3,4]. Expression of PPARγ has been shown in atherosclerotic lesions and macrophage foam cells, suggesting that PPARγ may affect atherosclerogenic processes [5,6]. The role of PPARγ in CAD could be mediated through its effects on adipocyte differentiation, lipid metabolism and inflammatory. The accumulation of lipids and extracellular matrices in the arterial intima elicits a local inflammatory response, leading to atherosclerogenic processes [7]. PPARγ agonists reduce triglyceride accumulation and clinically improve the outcome of atherosclerotic disease [8,9]. Diabetes mellitus is associated with an increased risk of developing atherosclerotic vascular disorders and cardiovascular disease [10,11]. The pathogenesis of CAD in diabetes is multifactorial in metabolic changes. The oxidative stress, glycoxidation, endothelial dysfunction, inflammation, and a diabetes-associated prothrombotic state have been implicated to play a role in the cardiovascular complications of diabetes [12]. Previous studies have shown that PPARγ agonist, pioglitazone improves endothelial dysfunction in patients with diagnosed T2DM and CAD [13,14].

Several mutations have been reported in the PPARγ gene. Pro12Ala has been reported to be associated with greater insulin sensitivity in childhood obesity and increased risk for diabetes [15]. The role of another variant, Pro115Gln polymorphisms, on the pathogenesis of obesity, type 2 diabetes, and related metabolic disorders was investigated in a Caucasian cohort, and no significant differences were found in lipoprotein metabolism and diabetes manifestation by comparing the different genotypes [16]. C161→T substitution at exon 6 of the PPARγ gene has been found to be associated with insulin resistance in Hispanic and non-Hispanic white women, considers to be a better predictor of fasting insulin levels and insulin resistance than P12A [17]. In the Metabolic Syndrome, the CC genotype was associated with severe lesion and the CT + TT with mild lesion of carotid artery, which implies that PPARγ C161→T may play an important role in carotid artery atherosclerotic [18]. However, the association between the PPARγ C161→T and the occurrence of CAD in the Chinese Han population with or without T2DM is not clarified.

In this study, we explored the PPARγC161→T substitution in well characterized hospital-based patients with CAD, T2DM, or CAD with T2DM, as well as healthy controls. The mechanism by which PPARγ C161→T alters the risk of severe vascular stenosis in the Chinese Han population was investigated from a genetic standpoint. Our results indicate that PPARγC161→T may be a predictor for risk of severe CAD in Chinese T2DM patients.

## Materials and methods

### Patients and protocol

We studied 556 kinless subjects of the Han nationality aged 70 years or less, including men (n = 339) and women (n = 217), consecutively admitted to the Zhongnan Hospital at Wuhan University and the Tongji Hospital at Huazhong University of Science and Technology from 2003 to 2007. This was the initial admission for the diagnosis of CAD for these patients. Diagnostic criteria for CAD was according to the ACC/AHA 2002 guidelines, and T2DM was according to the definition of diabetes advocated by the American Diabetes Association in 1997. All underwent angiography, and each angiogram was classified into two groups: having coronary lesion coronary angiography with less than 75% luminal stenosis or having at least one major epicardial coronary arteries with more than 75% luminal obstruction. The diagnosis from two cardiologists, based on coronary angiography, showed that of the 467 recruited patients, 78 patients had CAD, 86 patients had T2DM, and 303 patients had CAD combined with T2DM. Each patient's medical history was obtained using a questionnaire with standardized choices of answers to be checked during the interview. In this study period, patients did not use aspirin, insulin, oral hypoglycemic agents, or any other drug that could affect the lipid profile. The samples for DNA analysis were collected for each patient as previously described [19]. This study was approved by an external ethics committee, and written informed consent was obtained from each patient. A total of 89 healthy subjects were recruited in this study as controls.

### Biometric measurements

The height and weight were measured for each patient by a registered nurse who interviewed the patients to record the medical history. The body mass index (BMI) was obtained from the ratio of weight (kg) to height squared (m^2^). A BMI of 20-25 was considered normal, 25-30 over-weight, and >30 obese.

### Measurements of lipoproteins, apolipoproteins, blood glucose and serum insulin

The total cholesterol (TC), HDL-cholesterol (HDL-C) and triglyceride levels were measured in the hospital's Clinical Chemistry Department using standard enzymatic methods. The concentrations of apo A, apo B and Lp(a) were measured using a ELISA kit following manufacturer's instruction (Sigma). The LDL-cholesterol levels were calculated using the Friedewald formula. The concentrations of glucose and insulin were measured during a 75-g oral glucose tolerance test, and insulin resistance was estimated according to the homeostatic model [20] [(homeostasis model assessment-insulin resistance [HOMA-IR]) = fasting serum insulin × fasting blood glucose/22.5].

### Determination of the PPARγ exon 6 (C161→T) substitution

Whole blood, 5 ml samples with heparin, was collected. DNA was extracted from blood cells using a Whole Blood DNA Isolation Kit (Dojindo Molecular Technologies, Inc). The concentration of DNA recovered was expressed as UV spectro photometric OD260/OD280 ratios. The polymerase chain reaction (PCR) was used to detect the C161→T at exon 6 of the PPARγ gene as previously described by Meirhaeghe et al [21]. The forward primer was 5'-CAA GAC AAC CTG CTA CAA GC-3' and reverse primer was 5'-TCC TTG TAG ATC TCC TGC AG-3'. The amplification was performed in a 20 μl volume containing 200 ng DNA, 20 pmol of each primer, 2.0 mmol/l Mgcl_2_, 50 mmol/l KCl, 25 μmol/l dNTP and 1 Unit Taq polymerase. Samples were subjected to denaturing at 94°C for 5 min followed by 35 cycles of 94°C for 1 min, 57°C for 1 min, 72°C for 1 min. The final thermal cycle was at 72 °C for 5 min. The 200 bp PCR products were digested with an *EcoR *72I restriction enzyme and visualized by 8% agarose gel electrophoresis.

### Statistical analysis

SPSS 11.0 software was used to analyze the data. All the continuous variables are presented as mean ± SEM. The effects of genotypes were analysed and presented for all three genotype groups (CC, CT and TT). Since the number of the TT homozygous patients was small, we combined the TT and CT into a single "T" allele carrier group (CT+TT) to improve statistical power. We used a one-way ANOVA and a Student t test to evaluate relationships between the PPARγ genotypes and quantitative variables. To determine the correlations between the genotypes and other medical conditions including myocardial infarction, angina pectoris, diabetes mellitus and hypertension, a contingency table chi-square analysis was used to analyze the contribution of the polymorphism to the presence and severity of CAD. The Hardy-Weinberg equilibrium test was performed using the chi-square test. The statistical significance was defined as P < 0.05.

## Results

### The distribution of C161→T polymorphism between patients and the healthy controls

**Figure 1:**
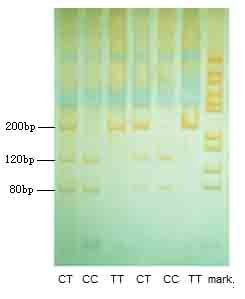
**Gene type analysis of PPARγ gene C161→T**. M: PGEM-7Z(+)HaeIIIDNA molecular marker

The length of exon 6 of PPAR gene was 249 bp, the DNA sequence of gene nt159-164 was CACGTG which was recognized by restriction endonucleotide enzyme E*coR *72I. The mutation C161→T eliminates an E*coR *72I recognition site and appears a polymorphism site. The genotypes of variants were analysed and presented for all three genotype groups CC (CACGTG) and CT/TT (CATGTG). In this study, polymerase chain reaction-restriction fragment length polymorphism was used to study the distribution of the PPARγ C161→T polymorphism. The primers used to amplify part of exon 6 of the PPAR gene were derived from the genomic sequence of the PPAR and used of these primers gave a PCR product of 200 bp. As shown in Figure 1, two fragments (120 bp and 80 bp) of PCR products were observed in wild-type. Only one fragment (200 bp) was seen in PPARγC161→T substitution, since the restriction site was eliminated by the C161→T transition. The genotypes were identified as CC, CT and TT. The most common allele has a C at nt 161, while the variant allele has a T at this position. The genotype distribution was in Hardy-Weinberg equilibrium in the control group (P = 0.97). However, no significant difference was observed in "T" allele frequency between all patient groups and healthy subjects. In the healthy group, "T" allele carries were 21.3%, and "C" allele carries were 78.7%. However the T allele carriers in the healthy control group were higher than those in CAD (19.8%), T2DM (20%), and CAD combined with T2DM (19.2%) groups (Table 1).

**Table 1:** Comparison of genotype distributions in CAD, T2DM and CAD with T2DM

	PPARγ C161→T genotypes			allele frequency	
		
Groups	CC	CT	TT	C	T
CAD (n = 78)	0.641(50)	0.321(25)	0.038(3)	0.802	0.198
T2DM (n = 86)	0.639(55)	0.314(27)	0.047(4)	0.796	0.20
CAD with T2DM (n = 303)	0.650(197)	0.314 (95)	0. 036(11)	0.808	0.192
Healthy control (n = 89)	0.618 (55)	0.337(30)	0.045(4)	0.787	0.213

No statistically significant difference was observed between the CAD patients and healthy subjects (*P *> 0.05)

### The C161→T polymorphism and severity of atherosclerosis and restenosis in CAD and CAD with T2DM patients

Since the lesions (luminal obstructions) in T2DM group patients were less than 50%, the relationship between C161→T polymorphism and severity of atherosclerosis and restenosis in T2MD patients was not shown in this study. The CAD and CAD plus T2DM patients have been divided into moderate and severe stenoses groups, according to the severity of stenosis in the major coronary arteries (modest group ≤75% but ≥ 50%, severe group ≥ 75% luminal obstructions). In the CAD group, simple X^2 ^comparison results showed that 33.1% of the 'T' allele carrier had the serious vessel disease and 38.1 % of the 'T' allele carriers had modest vessel disease. However, there was no significant difference between CC homozygotes and 'T' allele carriers (p = 0.695, Table 2). Data from the 303 CAD plus T2MD participants are presented in Table 3. All CAD plus T2MD patients had angiographically demonstrable coronary artery disease. A total of 155 patients had severe vessels stenosis (>75%), including 109 patients (70.3%) with the CC genotype, and 46 patients (29.7%) with the CT+TT genotype. A significant difference in severity of atherosclerosis and restenosis was found between patients with the CC genotype and patient bearing at least one T allele (the CT/TT genotypes) in CAD plus T2MD participants (OR = 1.22, 95%CI: 1.03-1.45, P = 0.019). This result indicates a significant association between the 161T polymorphism and severity of diseased vessels in CAD plus T2MD patients, but not in CAD patients.

**Table 2:** The correlation between PPARγ polymorphism and severity of atherosclerosis and restenosis in CAD group (%)

	PPARγ genotypes			
	
Groups	CC	CT	**TT **^#^	CT+TT*
Modest stenosis <75%	13 (61.9%)	7 (33.3%)	1 (4.8%)	8 (38.1%)
Severe stenosis >75%	38 (66.7%)	17 (29.8%)	2 (3.5%)	19 (33.3%)
Total (78)	51 (65.4%)	24 (30.8%)	3 (3.8%)	27 (34.6%)

Number of patients is presented in each subgroup with a row percentage in the bracket. # χ^2 ^= 0.113, P = 0.737, for comparisons among CC, CT genotypes. * χ^2 ^= 0.154, P = 0.695 for comparison between CC genotype and "T" allele carriers (CT+TT genotype).

**Table 3:** The correlation between PPARγ polymorphism and a severity of atherosclerosis and restenosis in CAD with T2DM group

	PPARγ genotypes			
	
Groups	CC	CT	**TT**^**#**^	CT+TT*
Modest stenosis<75%	85 (57.4%)	58 (39.2%)	5(3.4%)	63(42.6%)
Severe stenosis>75%	109(70.3%)	39(25.2%)	7(4.5%)	46(29.7%)
Total (303)	194(64%)	97(32%)	12(4%)	109(36%)

Number of patients is presented in each subgroup with a row percentage in the bracket. #χ^2 ^= 6.607, P = 0.010, for comparisons among CC, CT and TT genotypes. *χ^2 ^= 5.462, P = 0.019 for comparison between CC genotype and "T" allele carriers (CT+TT genotype). Taking CC genotype as the risk allele, the corresponding odds ratio with 95% confidential interval is 1.22 (1.03-1.45).

### The C161→T polymorphism and lipid metabolism

In the CAD (Table 4) and T2DM (Table 5) groups, no significant difference in lipid and blood glucose metabolism, including insulin resistance, was observed between PPARγ genotypes (CC versus CT/TT). However, there were significant differences in the levels of triglyceride and apoB in the CAD with T2DM group between CC genotype patients and T allele carriers (Table 6). The differences in apoB between genotypes remained significant after controlling for age, cigarette smoking status, and BMI, by a general factorial model of variance (*P=*0.032). Assessment-insulin resistance and blood glucose distributions showed no difference amongst all patients in the PPARγC161→T genotype group.

**Table 4:** Biometric, lipoprotein profile distributions, assessment-insulin resistance and blood glucose distributions in relation to the PPARγ C161→T genotypes in CAD patients

	PPARγ genotypes			
	
Groups	CC (n = 50)	CT (n = 25)	TT (n = 3)	CT+TT (n = 28)
Age	62.40 ± 12.04	67.75 ± 11.18	63.00 ± 8.73	66.80 ± 10.12
Weight (kg)	62.93 ± 9.59	58.23 ± 7.13	58.67 ± 7.51	58.32 ± 6.9 2
Height (m)	1.61 ± 0.07	1.63 ± 0.09	1.61 ± 0.06	1.63 ± 0.08
BMI (kg/m^2^)	22.48 ± 6.60	21.79 ± 3.14	23.01 ± 3.14	22.05 ± 3.06
TC (mmol/L)	4.37 ± 1.32	4.76 ± 1.36	3.82 ± 0.88	4.57 ± 1.31
Trig (mmol/L)	1.65 ± 0.89	1.51 ± 0.85	1.70 ± 0.82	1.55 ± 0.82
HDL (mmol/L)	1.21 ± 0.23	1.58 ± 0.62	1.13 ± 0.18	1.49 ± 0.59
LDL (mmol/L)	2.82 ± 0.69	2.77 ± 1.05	2.09 ± 0.60	2.63 ± 0.99
apoA1 (g/L)	1.29 ± 0.17	1.39 ± 0.22	1.27 ± 0.08	1.37 ± 0.20
apoB (g/L)	1.08 ± 0.11	0.87 ± 0.29	0.74 ± 0.28	0.85 ± 0.29
Lp(a) (mg/L) ^Δ^	1.90 ± 0.64	1.89 ± 0.58	1.25 ± 0.03	1.78 ± 0.58
FBS (mmol/L)	5.49 ± 0.77	5.78 ± 0.75	5.46 ± 1.06	5.71 ± 0.79
PPBS (mmol/L)	6.38 ± 1.22	8.17 ± 2.03	6.87 ± 0.28	7.87 ± 1.85
RI ^Δ^	1.08 ± 0.16	1.03 ± 0.21	1.12 ± 0.14	1.05 ± 0.19

No statistically significant difference was observed between the gene type groups (*P *> 0.05)

^Δ ^The measurement material were descriptive statistics of skewed distribution, and counted after logarithm

**Table 5:** Biometric, lipoprotein profile distributions, assessment-insulin resistance and blood glucose distributions in relation to the PPARγ C161→T genotypes in T2DM patients

	PARγ genotypes			
	
	CC(n = 55)	CT(n = 27)	TT(n = 4)	CT+TT(n = 31)
Age	58.13 ± 10.68	54.80 ± 11.05	56.50 ± 13.40	55.26 ± 12.21
Weight (kg)	66.10 ± 12.15	62.30 ± 9.06	69.38 ± 12.93	63.79 ± 10.02
Height (m)	1.65 ± 0.09	1.63 ± 0.07	1.66 ± 0.09	1.64 ± 0.07
BMI (kg/m^2^)	24.69 ± 4.65	23.04 ± 3.36	25.08 ± 4.67	23.62 ± 3.71
TC (mmol/L)	4.56 ± 0.89	4.42 ± 0.73	4.13 ± 0.34	4.36 ± 0.67
Trig (mmol/L)	1.93 ± 1.74	1.68 ± 1.02	1.77 ± 0.38	1.70 ± 0.92
HDL(mmol/L)	1.10 ± 0.37	1.27 ± 0.42	1.14 ± 0.27	1.25 ± 0.39
LDL (mmol/L)	2.77 ± 0.76	2.56 ± 0.70	2.49 ± 0.41	2.55 ± 0.64
apoA (g/L)	1.27 ± 0.19	1.39 ± 0.24	1.31 ± 0.13	1.36 ± 0.20
apoB (g/L)	1.14 ± 0.36	0.98 ± 0.16	1.03 ± 0.20	0.99 ± 0.18
Lp(a) (mg/L) ^Δ^	1.66 ± 0.64	1.67 ± 0.46	1.24 ± 0.04	1.53 ± 0.43
FBS (mmol/L)	9.58 ± 1.80	9.92 ± 2.35	10.31 ± 0.93	10.01 ± 2.11
PPBS (mmol/L)	13.38 ± 3.43	14.05 ± 4.22	15.07 ± 2.73	14.26 ± 3.
RI ^Δ^	1.84 ± 0.27	1.87 ± 0.25	1.77 ± 0.27	1.85 ± 0.25

No statistically significant difference was observed between the gene type groups (*P*>0.05)

^Δ ^The measurement material were descriptive statistics of skewed distribution, and counted after logarithm

**Table 6:** Biometric, lipoprotein profile distributions, assessment- insulin resistance and blood glucose distributions in relation to the PPARγ C161→T genotypes in CAD with T2DM patients

	PPARγ genotypes			
	
groups	CC (n = 197)	CT (n = 95)	TT (n = 11)	CT+TT (n = 106)
Age	62.64 ± 11.22	63.00 ± 12.87	66.00 ± 11.33	63.49 ± 12.61
Weight (kg)	64.35 ± 11.09 *	60.82 ± 9.16*	61.21 ± 7.31	61.38 ± 9.24
Height (m)	1.64 ± 0.09	1.63 ± 0.07	1.64 ± 0.07	1.63 ± 0.07
BMI (kg/m^2^)	23.94 ± 5.50	22.57 ± 3.62	24.06 ± 3.22	22.82 ± 3.58
TC (mmol/L)	4.66 ± 1.09	4.54 ± 1.17	4.19 ± 0.68	4.48 ± 1.11
Trig (mmol/L)	2.52 ± 1.59 *	1.68 ± 0.96 *	1.64 ± 0.46	1.67 ± 0.89*
HDL (mmol/L)	1.21 ± 0.38	1.22 ± 0.43	1.22 ± 0.24	1.22 ± 0.41
LDL (mmol/L)	2.74 ± 0.73	2.73 ± 0.91	2.38 ± 0.86	2.68 ± 0.90
apoA1 (g/L)	1.27 ± 0.19	1.25 ± 0.24	1.26 ± 0.10	1.25 ± 0.22
apoB (g/L)	1.02 ± 0.22*	0.94 ± 0.24*	0.94 ± 0.19	0.94 ± 0.23*
Lp(a) (mg/L) ^Δ^	1.82 ± 0.52	1.82 ± 0.53	1.32 ± 0.22	1.75 ± 0.53
FBS (mmol/L)	6.80 ± 2.26	7.32 ± 2.81	7.24 ± 2.49	7.23 ± 2.74
PPBS (mmol/L)	9.58 ± 3.90	10.73 ± 4.83	10.37 ± 4.36	10.67 ± 4.73
RI ^Δ^	1.42 ± 0.38	1.48 ± 0.42	1.34 ± 0.37	1.45 ± 0.41

* p < 0.05. P values were obtained by ANOVA F tests for comparisons among CC, CT and TT genotypes or Student t test for comparisons between CC genotype and T allele carriers (CT+TT genotypes).

^Δ ^The measurement material were descriptive statistics of skewed distribution, and counted after logarithm

## Discussion

PPARγ is critical transcription factor for the gene regulation of glucose and lipid metabolism. Therefore PPARγ is one of the potential candidate genes for the link between diabetes mellitus and CAD. A previous study showed an association between C161→T polymorphism in the PPARγ gene and a reduced risk of coronary artery disease in patients with and without diabetes in an Australian Caucasian cohort [19]. However, in a German study, an association between the C161→T polymorphism and the occurrence of coronary heart disease in patients with diabetes mellitus was lacking [22]. Recently, another study showed that PPARγ gene C161→T substitution was associated with reduced risk of coronary artery disease through modulation of pro-inflammatory cytokines, MMP-9 and TNF-α expression in a Chinese population [23]. Therefore, it has not clear if PPARγ gene C161→T substitution is associated with CAD in patients with or without T2MD. In this study, we investigated the correlation between the PPARγ C161→T and the occurrence of CAD in a Chinese Han population with or without T2DM, in order to determine a possible genetic marker to predict the development of CAD in patients with diabetes. We could not demonstrate a significant association of the PPARγ- C161→T genotype with CAD in the Chinese Han population with or without T2DM. However, PPARγ- C161→T markedly correlated with severity of atherosclerosis in patients with CAD with T2DM, which is through a mechanism underlying an altered lipid, but not glucose, metabolism.

Patients with diabetes mellitus have an increased risk of premature atherosclerosis [24]. T2DM causes more than two-fold increased incidence of myocardial infarction and CAD related death. Moreover, T2DM, but not coronary atherosclerosis was found to be an independent determinant of impaired mobility in the high risk population of patients with CAD who underwent angiography [25]. These data support the concept that diabetes is a major independent risk factor for CHD [26]. Both of CAD and T2DM have insulin resistance and hyperlipidemia, which produces detrimental effects on vasculature. High plasma glucose is known to be associated with the severity of coronary artery disease [27,28]. In this study, we evaluated the relationship between the PPARγ mutation and insulin resistance. Consistent with a previous report that studied a Brazilian population [29], we found that PPARγC161→T was not associated with insulin sensitivity and blood glucose level. However, a significant reduction of serum triglycerides was observed in the "T" allele carriers in patients with CAD and T2DM. Higher levels of apoB strongly correlated with elevated blood glucose levels and resulted in the occurrence of CAD. Moreover, high levels of apoB caused more inflammation and thrombosis in comparison with high levels of LDL. In this study, we also found that the level of apoB was higher in the CC genotype than the "T" allele carriers (CT+TT) in CAD combined with T2DM patients, indicating that PPARγC161→T reduces the risk of hyperlipidemia, and further diminishes the risk of severity of atherosclerotic vessels in patients with CAD and T2DM. Although CAD and T2DM are strongly associated with disorders of lipid metabolism, in our study, no significant differences in the levels of triglyceride and apoB were seen between the "C" and "T" allele carriers in the patients with CAD or T2DM, but a significant difference was observed in the patients with CAD and T2DM. This result suggests that the risk in disorders of lipid metabolism is increased when patients are classified as CAD plus T2DM. PPARγ C161→T may reduce this risk. Furthermore, severity of atherosclerosis and re-stenosis in CAD patients was not associated with genotypes, but "C" carriers in comparison with "T" carriers had a higher risk of severe atherosclerosis and re-stenosis in the patients with CAD and T2DM. This result further suggests that disorders of lipid metabolism may result in severe CAD. 70.5% patients with CAD and T2DM whose vascular stenosis was more than 75% had the CC gene type, thereby suggesting that diabetes can increase the severity of CAD.

In summary, we have found that the PPARγC161→T was associated with a reduced risk of severity of stenosis and lipid metabolism disorder in Chinese patients with CAD and T2DM. PPARγC161→T has a significant protective effect on atherogenesis in CAD and T2DM patients by modulating lipid metabolism. "T" allele carriers may transmit their genetic characteristics in the Han population to reduce the risk of severity of stenosis in patients with CAD and T2DM.

## Conclusion

PPARγC161→T genotypes weren't significantly associated with the risk of CAD, but were markedly correlated with severity of disease vessels in patients with CAD and T2DM. Furthermore, PPARγC161→T substitution was associated with an altered adipose, but not glucose metabolism. These results indicate that the PPARγ C161→T polymorphism may reduce the risk of severe atherogenesis by modulation of adipose metabolism, especially triglycerides and apoB, in Chinese patients with CAD and T2DM.

## Competing interests

The authors declare that they have no competing interests.

## Authors' contributions

J Wan supervised the project, designed and performed experiments, collected and analysed data and wrote the paper. S Xiong and J Xiao collected data. S Chao and Y Ma analysed data, discussed the results and commented on the manuscript. J Wang and S Roy interpreted the data and revised the manuscript. All authors read and approved the final version of the manuscript.
